# To block it, or not to block it?

**DOI:** 10.1007/s00432-017-2400-z

**Published:** 2017-03-27

**Authors:** Artur Wnorowski

**Affiliations:** Department of Biopharmacy, Medical University of Lublin, Collegium Pharmaceuticum, Chodzki 4a01a, 20-093 Lublin, Poland

To Editor,

In a recent issue of *Journal of Cancer Research and Clinical Oncology*, Coelho et al. (2017) reviewed preclinical and epidemiological reports regarding changes in cellular proliferation in response to β-adrenergic receptor (β-AR) ligands. The authors indicated that cancer cells commonly express both β_1_ and β_2_ adrenoceptors and that the agonist-mediated activation of these receptors leads to increased proliferation of cultured cells of different origin. Consequently, β-blockers (β-AR antagonists) were proposed as adjuvants for cancer management. This was supported by numerous pharmacoepidemiological studies indicating that cancer patients experience beneficial effects like prolonged survival or reduced risk of metastasis due to chronic β-blocker intake for cancer-unrelated heart conditions (Barron et al. 2011; Lemeshow et al. 2011).

As noted by the authors, the amount of data generated over time strongly supports the notion that blockage of β-ARs may constitute a novel therapeutic option for cancer. However, a non-negligible body of evidence indicates that opposite measures—i.e. activation of β-AR—may also be effective in suppressing the growth of cancer cells, at least in some cases (Strell et al. 2012). Thus, the purpose of this letter is to highlight the studies demonstrating antitumorigenic effects of β-AR activation, as opposed to β-blocker usage. To start with, high-grade brain tumors are characterized by low activity of adenylyl cyclase and/or depletion of cytoplasmic cAMP. Concomitantly, glioma and astrocytoma cell lines like A-172 and 1321N1 are highly susceptible to increased production of cAMP, which can be achieved by treatment with cAMP analogues, adenylyl cyclase activators or phosphodiesterase inhibitors (Chen et al. 1998; Toll et al. 2011). It is not surprising that agonists of β-AR can mimic the antiproliferative effects of the above-mentioned compounds, as canonical β-AR signaling pathway involves the G_s_-dependent activation of adenylyl cyclase and subsequent accumulation of cAMP (Toll et al. 2011). In our study carried out in 1321N1 cells, the level of cAMP induced by the β_2_-AR agonists like fenoterol and isoproterenol positively correlated with decreased mitogenesis (Fig. 1). Similarly, decreased cell growth was observed in response to β-AR agonists in nontumor breast MCF-10A cells (Bruzzone et al. 2014) and in breast cancer cell line MDA-MB-231 (Carie and Sebti 2007). In significance, Gargiulo et al. demonstrated that β_2_-AR knock-down led to increase of cell proliferation and migration of MCF-7 breast cancer cell line, indicating that β_2_-AR exerts an inhibitory tone towards cell growth and motility at basal conditions (Gargiulo et al. 2014). When the same cell line was engineered to overexpress β_2_-AR, significant drop in cell growth and migration was observed (Gargiulo et al. 2014). In line with this work, our unpublished data on rat C6 glioma cells indicate that siRNA-mediated depletion of β_2_-AR leads to significant increase in phosphoactive forms of AKT and ERK1/2. Taken together, expression level of the β_2_-AR receptor is an important factor shaping the proliferative capacity of cancer cells, and should be taken into account when studying the cellular responses to β_2_-AR ligands.

**Fig. 1:**
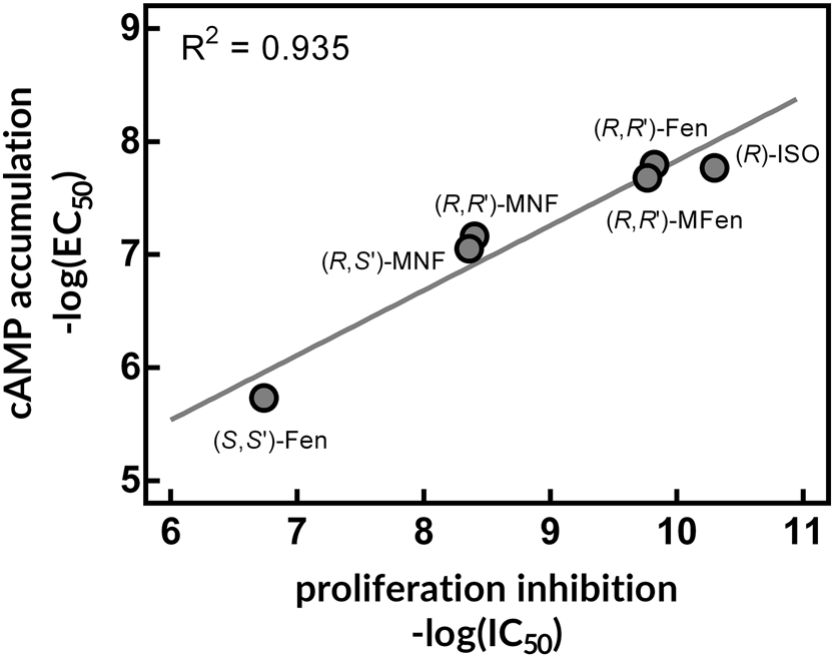
Antiproliferative effects of β_2_-AR agonists in 1321N1 astrocytoma cells. Selected β_2_-AR agonists were tested for the cAMP accumulation and for the [^3^H]thymidine incorporation in 1321N1 cells. Linear regression demonstrated that compounds more potent according to the capacity to induce cAMP production were also better inhibitors of thymidine incorporation. *ISO* isoproterenol, *Fen* fenoterol, *MFen* 4′-methoxyfenoterol, *MNF* 4′-methoxy-1-naphthylfenoterol. The graph was generated by plotting the data retrieved from the work of Toll et al. (2011)

Results from developmental studies also contribute to the knowledge on β-AR function in highly proliferative cells. Activation of β-ARs in murine embryonic pluripotent stem cells inhibits proliferation and cell cycle progression via cAMP and PKA (Sun et al. 2015). This is consistent with the finding that propranolol treatment in *Xenopus laevis* embryos encourages a neoplastic-like phenotype characterized by hyperpigmentation and increased melanocyte count (Sullivan and Levin 2016). We made compatible observations in human-derived UACC-647, M93-047 and UACC-903 melanoma cells: treatment with β_2_-AR agonists (isoproterenol, fenoterol) markedly delayed proliferation and invasiveness of the melanoma cells (Wnorowski et al. 2015). These functional responses were accompanied by drop in ERK1/2 phosphorylation. Pharmacological approach enabled us to map the signaling pathway responsible for the observed effects to β_2_-AR/G_s_/adenylyl cyclase/cAMP/PKA (Wnorowski et al. 2015). Comparable observations were made by Bruzzone and colleagues, who identified that isoproterenol mediates its antiproliferative actions by the activation of β_2_-AR/cAMP/PKA and boosts cell adhesion by triggering β_2_-AR/cAMP/EPAC in human breast cells (Bruzzone et al. 2014).

Plethora of epidemiological studies indicated that cancer patients benefit from β-blockers usage. For instance, population-based cohort study from Denmark showed that β-blocker treatment reduces risk of death in malignant melanoma patients (Lemeshow et al. 2011). Similarly, Watkins et al. reported longer median overall survival in ovarian cancer patients receiving nonselective β-blockers compared to nonusers (2015). However, some other reports failed to confirm the association between β-blocker use and decreased mortality following ovarian cancer diagnosis (Eskander et al. 2012; Johannesdottir et al. 2013). It has been speculated that some of the epidemiological reports on β-blockers can suffer from the immortal person-time bias (Schmidt and Schmidt 2016). This type of statistical bias arises when exposure to β-blocker occurs after the start of follow-up (i.e. after the cancer diagnosis). In such case, the time that patients have to survive between the start of follow-up to the initiation of β-blocker intake is referred to as “immortal”, as the patient needs to survive it to be classified as exposed to the drug. Inclusion of this immortal time leads to overestimation of the overall survival time in exposed group compared to nonusers (Suissa 2008). Thus, skew by an immortal time bias can constitute a probable explanation to discrepancies in retrospective pharmacoepidemiological studies on β-blocker effectiveness in ovarian cancer, and possibly in other cancer types (Weberpals et al. 2016).

## Conclusions

β-Blockers are commonly used drugs with well-defined pharmacokinetic and pharmacodynamic profiles and generally mild side effects (Hilal-Dandan et al. 2017). It would be greatly appreciated if these relatively inexpensive drugs were proven effective as supportive agents in standard chemotherapy or as standalone cancer prevention agents. Hopefully, ongoing clinical trials will confirm the feasibility of β-blockers usage in cancer management. However, in some types of cancer cells an opposite approach may be more suitable. Preclinical studies indicate that in some systems agonist-mediated activation of β-ARs efficiently suppresses cellular proliferation and, thus, is more beneficial than the treatment with β-blockers. We believe that there is an urgent need to understand why some cancer cells are susceptible to β-AR antagonists, while others are attenuated by agonists of β-ARs.

We fully agree with Coelho et al. that further studies are necessary to decipher the complexity of intracellular signaling triggered by different classes of β-AR ligands. When studying effects of such compounds, one should take into account the qualitative and quantitative variability in the composition of cellular signaling machinery, including the expression level and localization of adrenoceptors (Gargiulo et al. 2014) and downstream signaling proteins (Skalhegg and Tasken 2000) in the cells of interest. Availability of non-adrenergic receptors to oligomerize with β-ARs should be also acknowledged (Wnorowski and Jozwiak 2014). Moreover, single-dose experiments should be avoided in favor of dose-ranging studies, as cellular responses to β-AR ligands tend to follow biphasic, bell-shaped or U-shaped curves (Bruzzone et al. 2014; Wnorowski et al. 2015). With new data being consistently generated, we may ultimately be able to predict the cellular responses of a given cell type to agonists and antagonists of β-ARs and exploit this knowledge to produce desired cellular effect, hopefully in clinically relevant manner.
